# GPU-BSM: A GPU-Based Tool to Map Bisulfite-Treated Reads

**DOI:** 10.1371/journal.pone.0097277

**Published:** 2014-05-19

**Authors:** Andrea Manconi, Alessandro Orro, Emanuele Manca, Giuliano Armano, Luciano Milanesi

**Affiliations:** 1 Institute for Biomedical Technologies, National Research Council, Segrate (Mi), Italy; 2 Department of Electrical and Electronic Engineering, University of Cagliari, Cagliari (Ca), Italy; UCLA-DOE Institute for Genomics and Proteomics, United States of America

## Abstract

Cytosine DNA methylation is an epigenetic mark implicated in several biological processes. Bisulfite treatment of DNA is acknowledged as the gold standard technique to study methylation. This technique introduces changes in the genomic DNA by converting cytosines to uracils while 5-methylcytosines remain nonreactive. During PCR amplification 5-methylcytosines are amplified as cytosine, whereas uracils and thymines as thymine. To detect the methylation levels, reads treated with the bisulfite must be aligned against a reference genome. Mapping these reads to a reference genome represents a significant computational challenge mainly due to the increased search space and the loss of information introduced by the treatment. To deal with this computational challenge we devised GPU-BSM, a tool based on modern Graphics Processing Units. Graphics Processing Units are hardware accelerators that are increasingly being used successfully to accelerate general-purpose scientific applications. GPU-BSM is a tool able to map bisulfite-treated reads from whole genome bisulfite sequencing and reduced representation bisulfite sequencing, and to estimate methylation levels, with the goal of detecting methylation. Due to the massive parallelization obtained by exploiting graphics cards, GPU-BSM aligns bisulfite-treated reads faster than other cutting-edge solutions, while outperforming most of them in terms of unique mapped reads.

## Introduction

**Figure 1:**
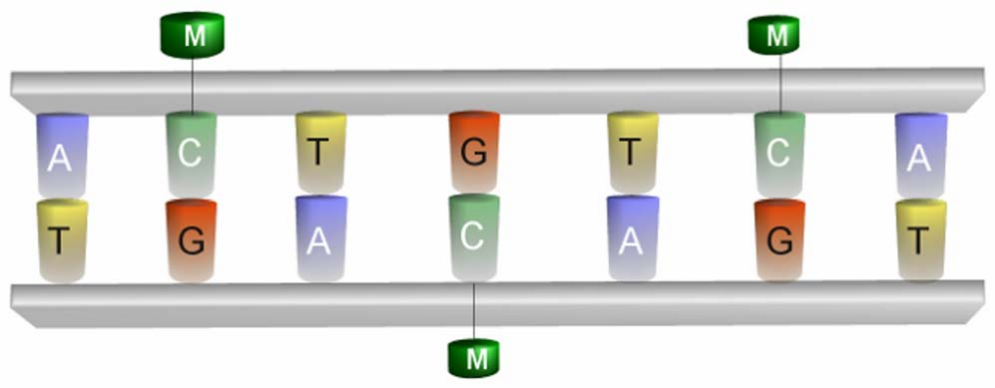
Cytosine DNA Methylation. Cytosine DNA methylation is a epigenetic mechanisms that affects gene expression. It involves the addition of a methyl group to the cytosine DNA nucleotides.

Regulation of gene expression is a very complex process controlled by multiple factors, including epigenetic ones. Epigenetics studies changes in gene expression that do not involve changes in the underlying DNA sequence [1]. Specifically, it refers to functionally relevant modifications that permit the genes of an organism to express themselves differently. Cytosine DNA methylation is a stable epigenetic mark that plays a very important role in several biological processes, including genomic imprinting, and is often responsible of phenotypic expressions (e.g., cancer) [2]. It involves the addition of a methyl group to the cytosine DNA nucleotides (see Figure 1). Mechanisms of epigenetic regulation include methylation at CpG islands in the promoter region of the gene. In many disease-causing processes gene promoter CpG islands acquire abnormal hypermethylation [3], which results in transcriptional silencing that can be inherited by daughter cells upon cell division. Three main approaches (i.e., endonuclease digestion, affinity enrichment and bisulfite conversion) [4] have been developed to analyze DNA methylation and various molecular biology techniques, as probe hybridization and sequencing, can be used to identify methylated cytosines in genomic DNAs treated with one of these approaches.

**Figure 2:**
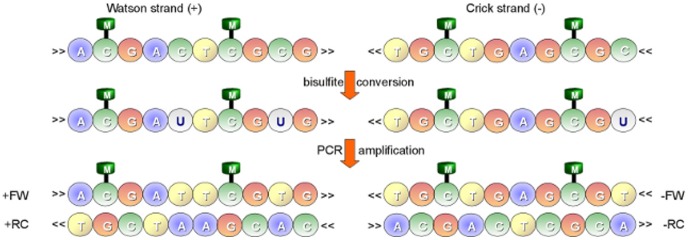
Bisulfite treatment. Two main type of libraries can be generated, directional and non-directional. As for directional libraries, a single amplification step is performed so that reads are related either to the forward (+FW) or to the reverse (-FW) direction of the bisulfite-treated sequence. Conversely, as for non-directional libraries, two amplification steps are performed, so that bisulfite reads may be related to four different directions of the bisulfite-treated sequence: forward Watson strand (+FW) and its reverse complement (+RC), forward Crick strand (-FW) and its reverse complement (-RC).

Bisulfite treatment of DNA [5] is considered the gold standard technique to study methylation. This technique introduces specific changes in the DNA sequence, depending on the methylation status of individual cytosine residues. Genomic DNA is modified by converting cytosines to uracils, while 5-methylcytosines remain nonreactive. In particular, during PCR amplification, only 5-methylcytosines are amplified as cytosine, whereas uracils and thymines are amplified as thymine. Two main protocols have been developed to construct bisulfite-treated libraries for high-throughput sequencing. These protocols, methylC-seq [6] and BS-seq [7], mainly differ in the PCR amplification procedure. In methylC-seq libraries are generated in a directional manner: a single amplification step is performed, so that reads are related to the forward (+FW) or to the reverse (-FW) direction of the bisulfite-treated sequence. Libraries generated using the methylC-seq protocol are termed directional. In BS-seq, two amplification steps are performed, so that bisulfite reads may be related to four different directions of the bisulfite-treated sequence: forward Watson strand (+FW) and its reverse complement (+RC), forward Crick strand (-FW) and its reverse complement (-RC) (see Figure 2). Libraries generated using the BS-seq protocol are termed non-directional.

The main limitation of whole-genome bisulfite sequencing (WGBS) is related to its cost, which is very high. Reduced representation bisulfite sequencing (RRBS) [8] is an alternative and cost-effective technique used to study methylation. In RRBS, DNA genome is first digested using specific restriction enzyme to enrich for CpGs. Then, the DNA fragments are size-selected and subsequently, as for WGBS, treated with bisulfite to be sequenced. Hence, in RRBS, only specific CpG-rich regions are considered.

**Figure 3:**
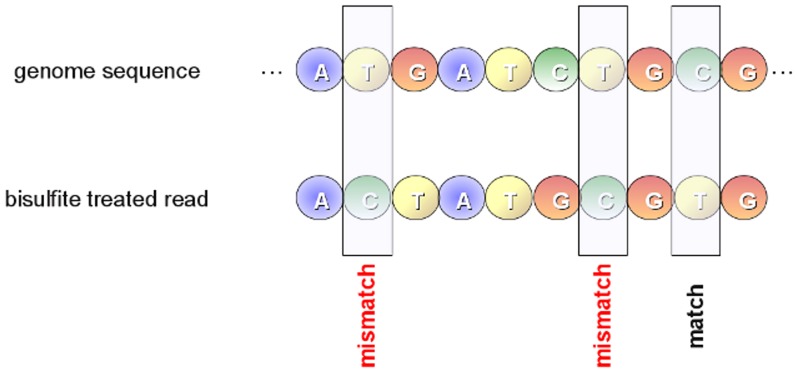
Asymmetric mapping. Due to the bisulfite treatment, unmethylated cytosines are converted to thymines during the PCR amplification. This conversion must be take into account during alignment by allowing an asymmetric mapping. A thymine in a read mapped to a cytosine in the reference genome sequence is considered as a match, whereas a thymine in the genome sequence mapped to a cytosine in a read is considered as a mismatch.

To calculate the methylation levels, bisulfite-treated reads are aligned against a reference genome. Mapping these reads to a reference genome represents a computational challenge mainly due to *i*) the increased search space and *ii*) the loss of information introduced by the bisulfite treatment. As for the former issue, considering that bisulfite affects only cytosines, non complementary Watson and Crick strands are generated. As previously highlighted, this implies that PCR amplification of both strands will produce up to four different strands and the bisulfite treated read can be derived from any of these strands. Moreover, the alignment process is further complicated by the asymmetric mapping between cytosines and thymines. In fact, a thymine in a read can be mapped to a cytosine in the reference genome, but the inverse is not allowed (see Figure 3). As for the latter issue, it should be pointed out that only a very small portion of cytosines is methylated in mammalian [9], making more difficult the alignment process along the reference genome.

Some tools have been proposed in the literature to address this mapping challenge. These tools can be divided in two classes, according to the strategy adopted to deal with the asymmetric mapping between cytosines and thymines. Tools belonging to the first class are specifically designed to perform alignments by allowing cytosines and thymines in the reads to match with cytosines in the reference sequence. By contrast, tools in the second class adopt an unbiased strategy that reduces the complexity of involved sequences converting cytosines to thymines. In so doing, sequences are represented with a simplified 3-letter nucleotide alphabet and alignments can be carried out with common and available short-read mapping tools. Alignments obtained exploiting this strategy must be post-processed to avoid those ambiguous and false positives. Tools in the first class provides the highest mapping efficiency. However, it should be observed that with the mapping strategy adopted by these tools, methylated read sequences will be aligned with greater efficiency than unmethylated ones. This means that tools in this class can overestimate methylation levels. By contrast, tools in the second class provide a slightly reduced mapping efficiency whereas alignment of reads is unaffected by their methylation state [10].

With no claim of being exhaustive, let us cite BSMAP/RRBSMAP [11] [12] (we point out that the latest release of RRBSMAP has been merged into BSMAP) and segemehl [13] as tools of the first class, and BS-Seeker/BS-Seeker2 [14] [15], Bismark [16], and BRAT-BW [17] are of the second class. BSMAP, applies to the reads a reverse bisulfite conversion, converting thymines to cytosines only at cytosine positions in the reference genome; then, it maps the masked reads to the genome. RRBSMAP, was the first tool specifically tailored for RRBS libraries. Based on suffix arrays, segemehl was the first tool able to take into account indels (insertions/deletions) in alignments of bisulfite-treated reads. Its high speed and accuracy are obtained by means of multi-threading, and with a very high memory consumption compared to those of others state-of-the-art tools. BS-Seeker performs a 3-letter alphabet reduction by converting cytosines to thymines on the FW reads and on both strands of the reference genome. Then, it uses the Bowtie [18] short-read alignment tool to map the converted FW reads against the converted Watson and Crick strands. In the event that reads are generated from the BS-seq protocol, a guanine to adenine conversion is performed on the RC of both reads and reference genome. Bowtie is then used to map the converted RC reads to the converted RC of the Watson and Crick strands. BS-Seeker runs in parallel the different instances of Bowtie. A final post-processing phase is performed to detect false positive alignments and methylation. BS-Seeker2 is an updated version of BS-Seeker that can also map reads from RRBS. Furthermore, BS-Seeker2 supports gapped global and local alignments with the newest multi-threading Bowtie2 [19] release. Bismark is an alternative tool able to map bisulfite-treated reads generated with both WGBS and RRBS. Similarly to BS-Seeker2, Bismark uses Bowtie2 and Bowtie to map preprocessed reads with and without indels supports respectively. Unlike from BS-Seeker2, Bismark does not support local alignments when used with Bowtie2. BRAT-BW uses the same strategy adopted by BS-Seeker and Bismark, while efficiently implementing the FM-index [20] in terms of memory occupancy. In general, due to the computational effort that may be required to cope with this mapping task, these tools present one or more implicitly or explicitly imposed constraints on the alignment process (e.g., number of mismatches, number of hits for reads, indels support). Table 1 reports a summarization of some features of the cited tools.

**Table 1:** Bisulfite-treated reads mapping tools.

tool	3-letter	mismatches	indels support	hits/reads	WGBS-RRBS
Bismark	Yes	Unlimited	Yes 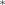	Unlimited	Yes
BSMAP	No	15	Yes	1000	Yes
BS-Seeker	Yes	3	No	2	only WGBS
BS-Seeker2	Yes	Unlimited	Yes 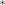	2	Yes
BRAT-BW	Yes	Unlimited	No	Unlimited	only WGBS
segemehl	No	Unlimited	Yes	Unlimited	only WGBS

Some bisulfite-treated reads mapping tools listed according to some relevant features. The second columns indicates whether the corresponding tool adopts a 3-letter conversion strategy. The third column reports the maximum number of mismatches allowed for the read. The fourth column reports whether the corresponding tool supports gapped alignments. The fifth column reports the maximum number of hits allowed for a read. The sixth column reports whether the corresponding tool supports WGBS and RRBS protocols.

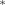
Using Bowtie2.

In this work, we present GPU-BSM (standing for GPU-BiSulfite reads Mapping), an accurate and very fast tool devised to map bisulfite-treated reads and to estimate methylation levels. Written in Python, GPU-BSM exploits the 3-letter nucleotide alphabet reduction strategy and it is mainly based on SOAP3-dp [21], a short-read mapping tool able to take advantage of the computational power of modern Graphics Processing Units (GPU). GPU-BSM has been designed to support ungapped and gapped (global and local) alignment with libraries generated with both WGBS and RRBS. Currently, GPU-BSM can be run parallelized on up to 4 different GPU cards. The massive parallelization obtained by means of GPUs enables GPU-BSM to map bisulfite-treated reads without imposing stringent limitations on the alignment process.

## Methods

Based on the 3-letter nucleotide alphabet reduction strategy, GPU-BSM implements an approach similar to the one adopted by similar tools as BS-Seeker, Bismark, and BRAT-BW. In particular, similarly to other tools, GPU-BSM uses a third-part short-read mapper (i.e., SOAP3-dp) to align 3-letter converted reads. In the following of this section, we first give a short introduction to GPUs and to existing state-of-the-art short-read mapping tools. Then, we present our strategy, devised to deal with the bisulfite-treated reads mapping problem. Subsequently, we discuss about the adopted alignment constraints. Finally, we briefly resume the hardware and software equipment required to use GPU-BSM.

### GPU

**Figure 4:**
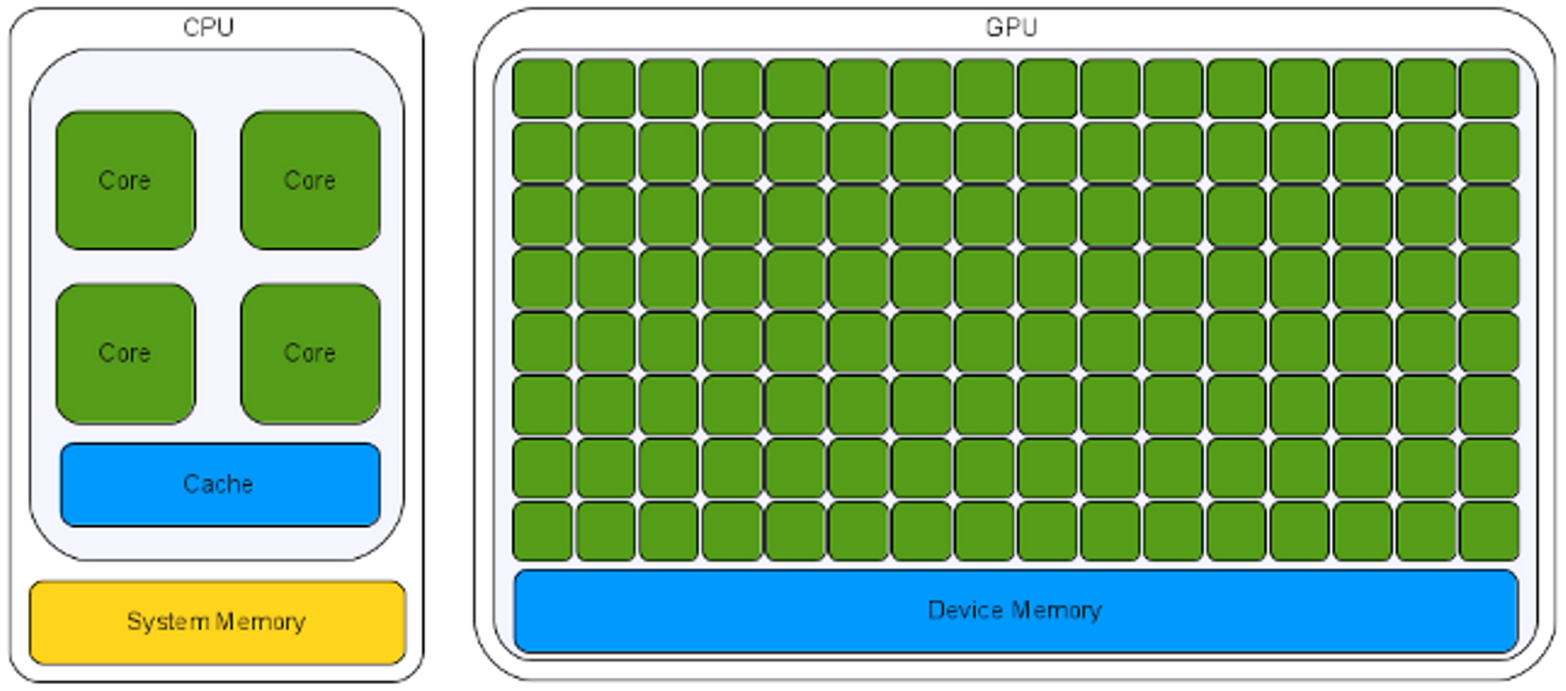
Multi-core and many-core processors. Multi-core processors as CPUs are devices composed of few cores with lots of cache memory able to handle a few software threads at a time. Conversely, many-core processors as GPUs are devices equipped with hundreds of cores able to handle thousands of threads simultaneously.

GPUs are hardware accelerators that are increasingly used to deal with computationally intensive algorithms. From an architectural perspective, GPUs are very different from traditional CPUs. Indeed, the latter are devices composed of few cores with lots of cache memory able to handle a few software threads at a time. Conversely, the former are devices equipped with hundreds of cores able to handle thousands of threads simultaneously, so that a very high level of parallelism (see Figure 4) can be reached. GPGPU (General Purpose Computing on Graphics Processing Units) is a methodology for high-performance computing that combines CPUs with GPUs to deal with data parallel and throughput intensive algorithms. As CPUs are more effective than GPUs for serial processing, they are used to perform serial parts of the algorithm, whereas GPUs are used to perform parts of the algorithm where processing of large blocks of data is done in parallel. The main disadvantage of using GPUs is related with the effort required to code algorithms. To take advantage of the GPU technology, algorithms must be coded to reflect the architecture of these hardware accelerators. Incorporating GPU support into existing codes is very difficult and typically requires significant changes of the code and of the algorithm.

In the NVIDIA GPU-based architecture, parallelization is obtained through the execution of tasks in a number of stream processors or CUDA cores. Cores are grouped in multiprocessors that execute in parallel. A CUDA core executes a floating point or integer instruction per clock cycle for a thread and all cores in a streaming multiprocessor execute the same instruction at the same time. The code is executed in groups of threads called warps. Device memory access takes a very long time due to the very long memory latency.

**Figure 5:**
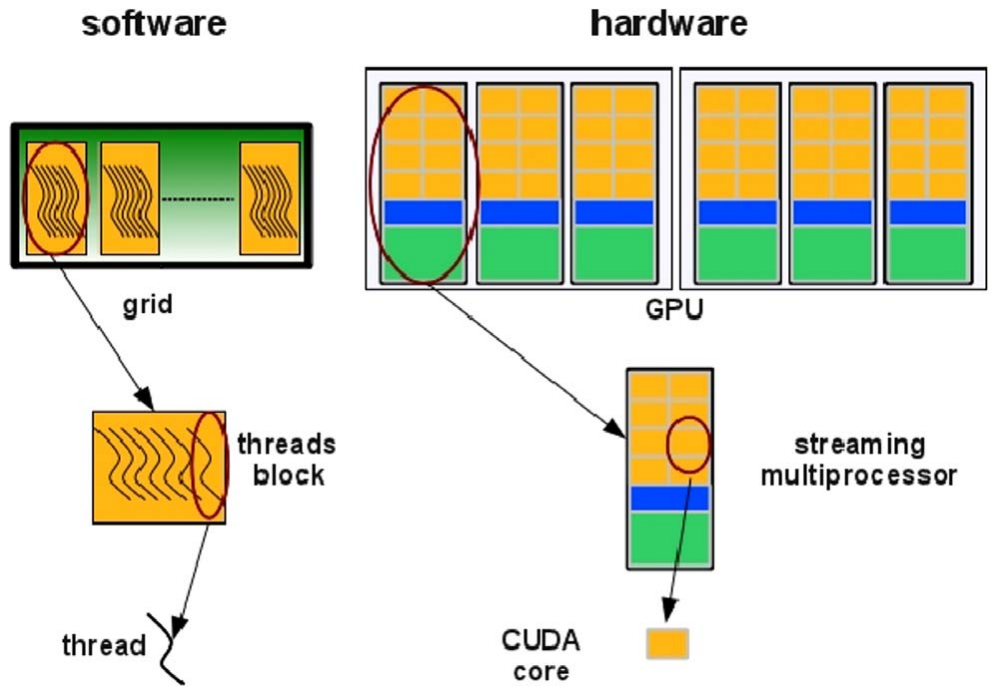
CUDA execution model. Threads are grouped in blocks in a grid. Each thread has a private memory and runs in parallel with the others in the same block.

The parallel programming model of the CUDA architecture provides a set of API that allows programmers to access the underlying hardware infrastructure and to exploit the fine-grained and coarse-grained parallelism of data and tasks. Summarizing, the CUDA execution model (see Figure 5) can be described as follow: the GPU creates an instance of the kernel program that is made of a set of threads grouped in blocks in a grid. Each thread has a unique ID within its block and a private memory and registers, and runs in parallel with others threads of the same block. All threads in a block execute concurrently and cooperatively by sharing memory and exchanging data. A block, identified by a unique ID within the block grid, can execute the same kernel program with different data that are read/written from a global shared memory. Each block in the grid is assigned to a streaming multiprocessor in a cyclical manner.

### Short-read mapping tools

Several tools have been devised to perform short-read mappings. Without claiming to be exhaustive, let us cite some of the most popular solutions, i.e. MAQ [22], RMAP [23], [24], Bowtie, BWA [25], CloudBurst [26], and SHRiMP2 [27], [28]. MAQ maps short sequence reads to a reference genome by calculating the probability of a read alignment to be correct, and consensus genotype calling with a model that incorporates correlated errors and diploid sampling. It supports gapped alignment and can align reads up to 128 bp. RMAP uses quality scores to provide accurate ungapped alignments. It exploits a first criterion based on a simple count of mismatches and a second criterion making use of the base-call quality scores. Bowtie is a memory-efficient short-read aligner that exploits the Burrows-Wheeler Transform (BWT) to index the genome allowing only ungapped alignments. BWA is another tool that exploits the BWT to index the reference sequences. Unlike from Bowtie, it can also provide gapped alignments. CloudBurst is a parallel seed-and-extend read-mapping tool able to align reads with a specified number of differences, including both mismatches and indels. It exploits the open-source Hadoop [29] implementation of MapReduce [30] to parallelize the execution using multiple computing nodes. SHRiMP2 exploits specialized vector computing hardware to speed-up the Smith-Waterman [31] dynamic programming algorithm. It is a multi-core short-read mapping tool that enables the alignment of reads with extensive polymorphism and sequencing errors. A comparative study aimed at assessing the accuracy and the runtime performance of different cutting-edge next-generation sequencing read alignment tools highlighted that among all SOAP2 [32] was the one that showed the higher accuracy [33]. Exhaustive review of the tools cited above can be found in [34].

In general, the mentioned solutions exploit some heuristics to find a good compromise between accuracy and running time. Recently, GPU-based solutions have been proposed to cope with different bioinformatics problems [35]–[38]. GPUs have also been exploited to cope with the exponentially increasing throughput of next-generation sequencing. In particular, the computational power of these hardware accelerators is helping researchers to speed the short-read mapping process without compromising accuracy. Lately, the GPU-based short-read mapping tools Barracuda [39], CUSHAW [40], SOAP3 [41] and SOAP3-dp have been proposed to the scientific community. Experimental results show that SOAP3, which is the GPU evolution of SOAP2, outperforms the popular tools BWA and Bowtie. When tested to align millions of 100-bp read pairs against the human genome, it resulted at least 7.5 times faster than BWA, and 20 times faster than Bowtie. Moreover, SOAP3 does not exploit heuristics and it is able to align correctly slightly more reads than BWA and Bowtie. SOAP3 is able to align a read to a reference sequence with up to four mismatches while it does not support gapped alignments. Lately, the SOAP3 research team released SOAP3-dp, a new version of the aligner that exploits dynamic programming to support gapped global and local alignments. Compared with BWA, Bowtie2 [19], SeqAlto [42], GEM [43], and the previously mentioned GPU-based aligners, SOAP3-dp is two to tens of times faster, while maintaining the highest sensitivity and lowest false discovery rate on Illumina reads with different lengths.

### The implemented strategy

Reads alignment may be very expensive in terms of both computing time and exploited hardware resources. Modern short-read mapping tools try to speed the alignment *i*) by parallelizing the overall process, and *ii*) by using ad-hoc heuristics. Parallelization could considerably accelerate the alignment, but it is often limited by the available hardware resources (i.e., CPU cores and memory). On the other hand, the adoption of heuristics may degrade sensitivity and affect the quality of the final results. As already pointed out, the computational challenge is heightened in the process to map bisulfite-treated reads, in which to map a read two or four different alignments must be performed according to the used protocol. Massive parallelization that may be obtained exploiting GPUs has been successfully used to address the short-read mapping problem and we deem that it may be exploited to address also the mapping of bisulfite-treated reads. In fact, GPU-BSM uses the GPU-based SOAP3-dp mapping tool to align bisulfite-treated reads.

Initially, GPU-BSM creates two sequences from the forward genomic strand. The first sequence is obtained by converting cytosines to thymines, whereas the second sequence is obtained by converting guanines to adenines. These sequences are created differently, depending on the sequencing technique used to generate the analyzed library. As for WGBS libraries, sequences are created from the original forward genomic strand, whereas for RRBS libraries they are created from a modified version to take into account the sequencing parameters. In particular, GPU-BSM modifies the genomic strand masking those DNA fragments that do not meet the sequencing parameters. In so doing, GPU-BSM notably improves the computing time required to align RRBS libraries.

**Figure 6:**
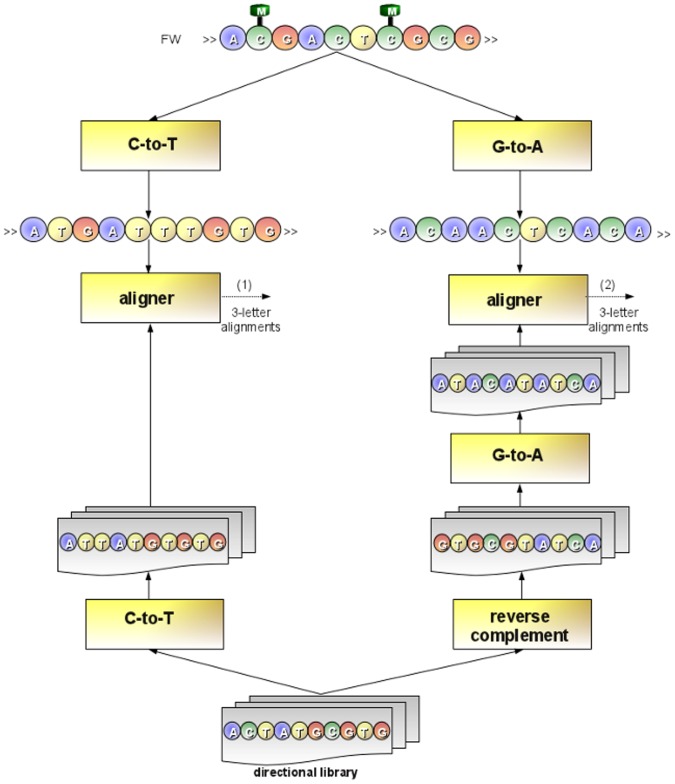
Mapping directional reads. To map directional reads, GPU-BSM performs two different alignments. As for the former alignment, GPU-BSM maps the reads of the library against the forward strand of the reference genome, after that cytosines have been converted to thymines in all sequences. As for the latter alignment, GPU-BSM maps the reverse complement of the reads against the forward strand of the reference genome, after that guanines have been converted to adenines in all sequences. Finally, all 3-letter alignments obtained for a read (i.e., outputs (1) and (2) in the figure) will be post-processed with the aim to detect and remove those ambiguous and false positives.

**Figure 7:**
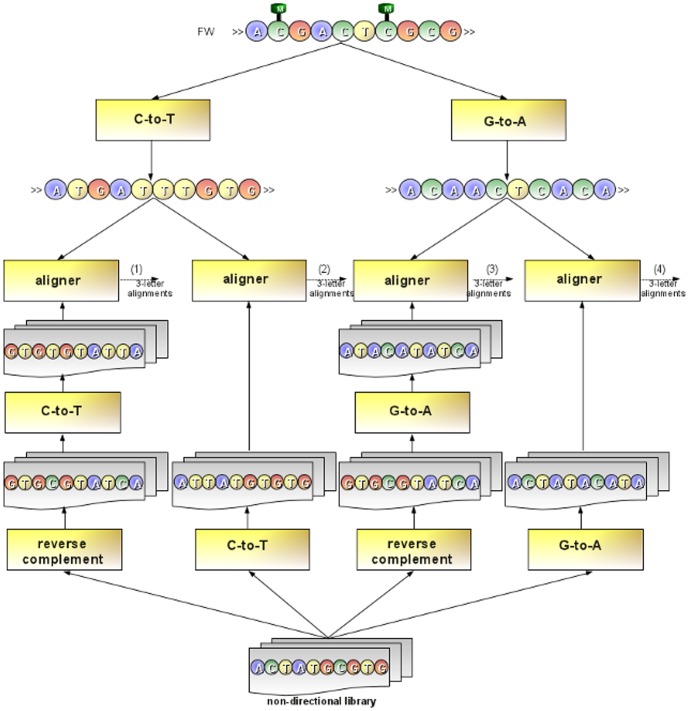
Mapping non-directional reads. To map non-directional reads, GPU-BSM performs four different alignments. The figure shows that two additional alignments are performed with respect to ones reported in Fig. 6 for directional reads. As for the first additional alignment, GPU-BSM maps the reads of the library against the forward strand of the reference genome after that guanines have been converted to adenines in all sequences. As for the second alignment, GPU-BSM maps the reverse complement of the reads of the library against the forward strand of the reference genome after that cytosines have been converted to adenines in all sequences. Finally, all 3-letter alignments obtained for a read (i.e., outputs (1), (2), (3) and (4) in the figure) will be post-processed with the aim to detect and remove those ambiguous and false positives.

**Figure 8:**
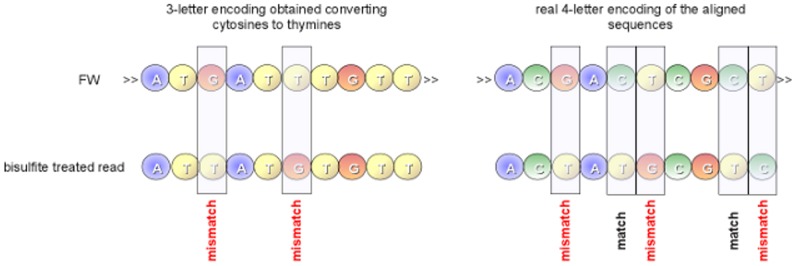
False positive alignments. GPU-BSM aligns reads exploiting a reduced 3-letter nucleotide alphabet. Alignments obtained using this encoding must be processed to look for false positives; i.e., those alignments that in the actual 4-letter nucleotide alphabet do not meet the alignment constraints imposed by the user. A typical case is represented in this figure. A two mismatches alignment obtained with the 3-letter encoding is reported on the left side. The same alignment, reported on the right of the figure with 4-letter nucleotide alphabet, shows three mismatches.

Directional and non-directional libraries are treated differently. To map reads from a directional library, GPU-BSM performs two different alignments using SOAP3-dp. The first alignment is obtained by converting cytosines to thymines in the reads and then aligning them to the first sequence. The second is obtained by converting guanines to adenines in the reverse complement of the reads and then aligning them to the second sequence (see Figure 6). To map reads from a non-directional library, GPU-BSM performs four different alignments. In addition to the alignments performed for a directional library, GPU-BSM uses SOAP3-dp to map the reverse complement of the reads with cytosines converted to thymines to the first sequence, and the reverse complement of the reads with guanines converted to adenines to the second sequence. Then, GPU-BSM analyzes the mapped reads, detecting and removing ambiguous reads and those that are in fact false positives (see Figure 7). We consider ambiguous those reads for which *i*) a best match exists for at least two of two/four alignments performed according to the exploited library or *ii*) at least two best hits exist for a single alignment. However, users interested in these mappings can disable this filtering option. To detect false positives, GPU-BSM calculates the number of mismatches of the mapped reads using the 4-letter nucleotide alphabet. Let us recall that, due to the bisulfite treatment, a thymine in a read can be aligned to a cytosine in the reference sequence. Similarly, a guanine in the reverse complement of a read can be aligned to an adenine in the reference sequence (see Figure 8).

To take advantage of multiple GPUs, GPU-BSM automatically runs in parallel the two (four) different alignments for directional (non-directional) libraries. In the current release, GPU-BSM performs alignments on up to four GPUs. In particular, it uses up to two GPU cards to perform two different alignments required for reads of directional libraries, whereas it uses up to four cards to perform four different alignments required for reads of non-directional libraries. For machine equipped with a single GPU card, GPU-BSM sequentially performs the different alignments.

### Tool settings

Depending on whether dynamic programming is enabled or not, SOAP3-dp will generate gapped or ungapped alignments. When dynamic programming is enabled, SOAP3-dp performs the alignment in two steps. In the first step, it uses SOAP3 to look for ungapped alignments that meet a given constraint on the allowed number of mismatches. Up to 4 mismatches are allowed for this step and no heuristic is used. In the second step, it exploits dynamic programming to look for gapped alignments. A score threshold defines when to use dynamic programming. It is also possible to skip the first step with the aim to align all reads exploiting the dynamic programming reducing the computing time. By default, in the first step SOAP3-dp allows up to two mismatches to speed-up the overall alignment process. However, GPU-BSM uses SOAP3-dp to aligns reads with up to four mismatches when it looks for ungapped alignments, whereas it does not allow mismatches in the first step when used to look for gapped alignments. It should be pointed out that this constraint refers to the number of mismatches allowed in the alignment when both read and genome are converted using the 3-letter nucleotide alphabet. Users can change this value in GPU-BSM as well as disable ungapped alignments. By default, GPU-BSM generates up to 2 alignments for a read. Users can easily modify this value to decrease, increase, or avoid the upper limit to the alignments that may be found for each sequence. By default, GPU-BSM analyzes only the unique best alignments found by SOAP3-dp. However, GPU-BSM also permits to analyze all valid alignments or all best alignments obtained by SOAP3-dp.

### Hardware and Software Requirements

GPU-BSM works on linux based systems, equipped with a custom installation of Python (release
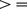
2.7.3) and with a CUDA enabled GPU-card. We tested it on two families of NVIDIA GPU cards. In particular tests have been carried out on the NVIDIA FERMI architecture based GTX 480 card, and on the NVIDIA Kepler architecture based k10 and k20c cards. Currently, SOAP3-dp can be run on the CUDA-4.2 and CUDA-5.5 releases. As SOAP3-dp has been successfully deployed on some cloud computing services (e.g., Amazon EC2, NIH BioWulf and Tianhe-1A) it is also possible to use our tool on them.

## Results

Experiments have been designed to assess the performances of GPU-BSM to map WGBS and RRBS libraries with both synthetic and real data. In this section, we first introduce experiments on synthetic data mainly aimed at assessing the reliability of GPU-BSM. Then, we present evaluation results on real data. Finally, we briefly resume the hardware and software configuration used for experiments.

### Performance evaluation on synthetic data

Synthetic WGBS and RRBS libraries have been generated with the Sherman bisulfite-read simulator (http://www.bioinformatics.babraham.ac.uk/projects/sherman/). For our experiments, we used libraries of different reads length. In particular, libraries with reads length of 75 and 120 bp have been generated. Each library consisted of 250 thousands of reads generated from the build 37.3 of the human genome with a uniform bisulfite conversion rate of 50% on both strands. Libraries have been generated simulating the sequencing error rate from 0% to 6% in increments of 2%. So, we generated sixteen libraries: eight synthetic WGBS libraries and eight synthetic RRBS libraries. Specifically, for both WGBS and RRBS, we generated four libraries for reads of length of 75 bp and four libraries for reads of length 120 bp with simulated sequencing errors of 0%, 2%, 4% and 6%, respectively. As for RRBS libraries, we performed an in-silico MspI digestion on the build 37.3, and selected 40–500 bp fragments.

Sherman simulates sequencing errors using an error rate curve that follows an exponential decay model with the aim to mimic real data. In this way, it will be most likely that the simulated errors are in bases towards the 3′ end rather than in bases towards the 5′ end.

As for WGBS libraries, GPU-BSM has been compared with Bismark, BSMAP, BS-Seeker2 and segemehl, whereas for RRBS libraries only with Bismark, BSMAP and BS-Seeker2 as segemehl does not support this type of data. To provide an accurate comparison with the other tools, experiments were performed to assess the reliability of GPU-BSM to look for ungapped and gapped alignments. In particular, as for gapped alignments, we separately assessed the performance of GPU-BSM when used to look for global and local alignments. Bismark and BS-Seeker2 have been used with Bowtie to look for ungapped alignments, and with Bowtie2 to look for gapped alignments. BS-Seeker2 with Bowtie2 has been run to look for gapped global and local alignments. BSMAP and segemehl look for gapped global alignments and do not permit to enable or disable this feature.

Tools compared in this work implement different algorithms that do not allow to perform experiments using the same constraints. Then, experiments have been performed setting parameters with the aim to obtain more accurate alignments according to the analyzed library (see Table 2). In particular, tools have been run to look for alignments with up to five mismatches. It should be pointed out that Bismark and segemehl do not permit to set the number of mismatches to be allowed; they permit to set the number of mismatches in the seed. Then, in order not to overestimate the performance of these tools, we analyzed their alignments without taking into account those obtained with more than five mismatches.

**Table 2:** Tool settings used to map synthetic reads.

tool		
GPU-BSM 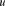	WGBS	-m 5 –ungapped -l 1
	RRBS	-m 5 –ungapped -l 1 -R -d C-CGG
GPU-BSM 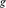	WGBS	-m 5 –e2e -l 1
	RRBS	-m 5 –e2e -l 1 -R -d C-CGG
GPU-BSM 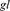	WGBS	-m 5 -l 1
	RRBS	-m 5 -l 1 -R -d C-CGG
Bismark 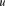	WGBS	-q –directional
	RRBS	-q –directional
Bismark 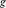	WGBS	-q –directional –bowtie2
	RRBS	-q –directional –bowtie2
BS-Seeker2 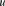	WGBS	-m 5 –aligner = bowtie -f sam
	RRBS	-m 5 –aligner = bowtie -f sam -r -c C-CGG -L 40 -U 500
BS-Seeker2 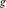	WGBS	-m 5 –aligner = bowtie2 –bt2–end-to-end -f sam
	RRBS	-m 5 –aligner = bowtie2 –bt2–end-to-end -r -c C-CGG -L 40 -U 500
BS-Seeker2 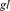	WGBS	-m 5 –aligner = bowtie2 -f sam
	RRBS	-m 5 –aligner = bowtie2 -r -c C-CGG -L 40 -U 500
segemehl	WGBS	-D 0 -F 1 -H 1
	RRBS	not supported
BSMAP	WGBS	-v 5 -w 2 -r 0
	RRBS	-v 5 -w 2 -r 0 -D C-CGG

Tool settings used to map reads of synthetic libraries. Default settings have been used for not specified parameters.

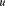
Ungapped alignments. In these experiments Bismark and BS-Seeker2 are used with Bowtie.

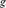
Gapped alignments. In these experiments Bismark and BS-Seeker2 are used with Bowtie2.

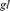
Gapped local alignments. In these experiments BS-Seeker2 is used with Bowtie2.

Very accurate tools will exhibit high precision and high recall (sensitivity). Then, with the goal of providing a rigorous comparison among the tools, we compared the performances of the analyzed tools in terms of unique best mapped reads, precision, and F1. Defined as the harmonic mean between precision *(p)* and recall *(r)*, F1 is a measure that weights equally both metrics. It penalizes systems with a mediocre performance of precision or sensitivity with respect to those that exhibit good performance on both metrics.

#### Performance evaluation on WGBS libraries

**Figure 9:**
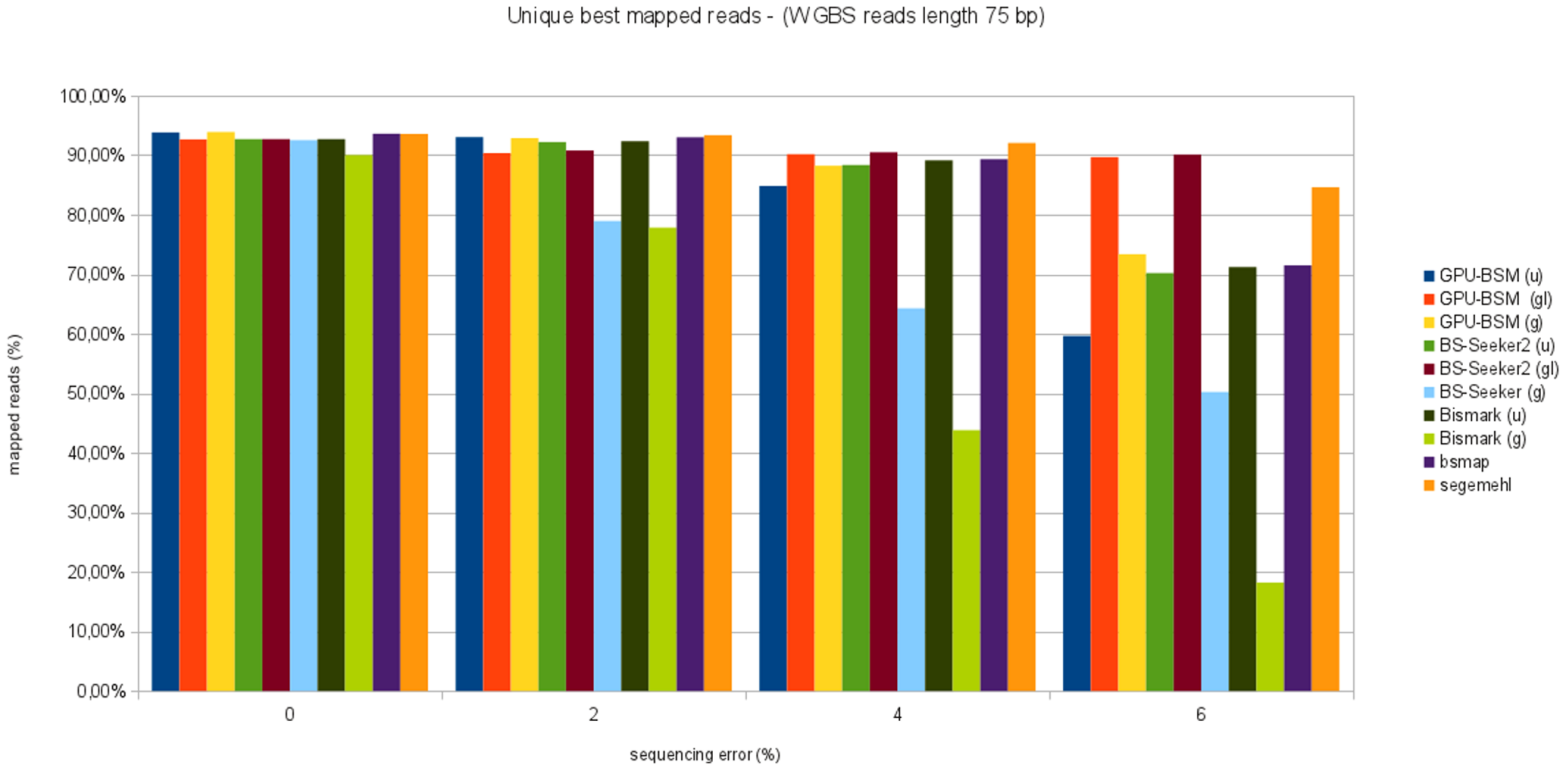
Unique best mapped reads for WGBS libraries with reads length of 75 bp. The graph represents the percentage of unique best mapped reads obtained for each tool as function of the sequencing error for WGBS synthetic libraries with reads length of 75 bp.

**Figure 10:**
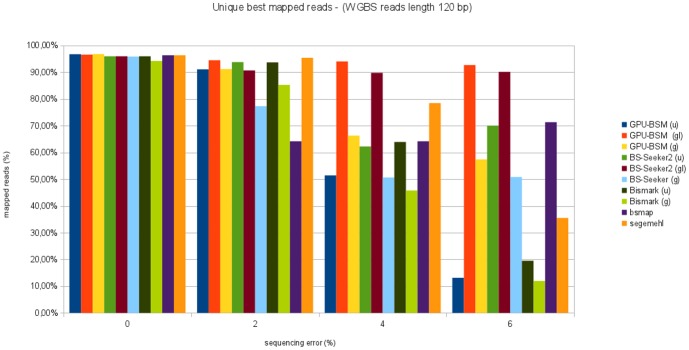
Unique best mapped reads for WGBS libraries with reads length of 120 bp. The graph represents the percentage of unique best mapped reads obtained for each tool as function of the sequencing error for WGBS synthetic libraries with reads length of 120 bp.

Figures 9 and 10 show the percentage of unique best mapped reads as function of sequencing error for WGBS libraries. In almost all cases GPU-BSM and BS-Seeker2, both run to support local alignments, have been able to map more reads than the other tools. GPU-BSM, when run supporting gapped global alignments was the second tool able to map more reads than the other ones for almost all simulated sequencing errors. In particular, GPU-BSM outperforms the other tools that adopt the same unbiased strategy. As for ungapped alignments, the number of reads mapped by GPU-BSM is comparable with those of Bismark and BS-Seeker2 for simulated sequencing error up to 2%.

The analysis of precision (see Table 3 and Table 4) shows that for local alignments GPU-BSM is more accurate than BS-Seeker2. As for gapped alignments, BSMAP and segemehl outperform the other tools, whereas for ungapped alignments Bismark and BS-Seeker2 are slightly more accurate than GPU-BSM.

**Table 3:** Precision for WGBS libraries with reads length of 75 bp.

	simulated sequencing error			
tool	0%	2%	4%	6%
GPU-BSM 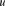	93.75%	92.91%	84.48%	58.74%
GPU-BSM 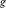	93.84%	92.68%	87.89%	72.75%
GPU-BSM 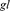	92.57%	89.92%	88.64%	89.09%
Bismark 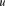	92.69%	92.30%	88.99%	70.86%
Bismark 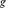	90.01%	77.75%	43.50%	17.85%
BSMAP	93.59%	92.96%	89.21%	71.08%
BS-Seeker2 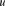	92.69%	92.16%	89.36%	88.73%
BS-Seeker2 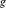	92.52%	78.60%	63.41%	49.28%
BS-Seeker2 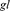	92.69%	90.08%	89.36%	88.73%
segemehl	93.57%	93.30%	91.96%	84.41%

Table reports precision varying the sequencing error from 0% to 6% for 250 thousands of 75 bp reads mapped against the build 37.3 of the human genome.

**Table 4:** Precision for WGBS libraries with reads length of 120 bp.

	simulated sequencing error			
tool	0%	2%	4%	6%
GPU-BSM 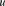	99.39%	99.16%	98.79%	98.56%
GPU-BSM 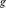	99.35%	99.09%	99.08%	99.25%
GPU-BSM 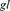	99.32%	98.62%	98.29%	98.49%
Bismark 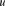	100%	99.78%	99.57%	98.32%
Bismark 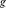	100%	99.87%	99.67%	97.76%
BSMAP	100%	99.45%	98.75%	98.07%
BS-Seeker2 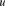	100%	99.67%	99.65%	98.36%
BS-Seeker2 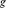	100%	98.61%	98.65%	94.76%
BS-Seeker2 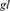	100%	95.41%	96.12%	86.55%
segemehl	100%	99.72%	99.50%	99.30%

Table reports precision varying the sequencing error from 0% to 6% for 250 thousands of 120 bp reads mapped against the build 37.3 of the human genome.

**Figure 11:**
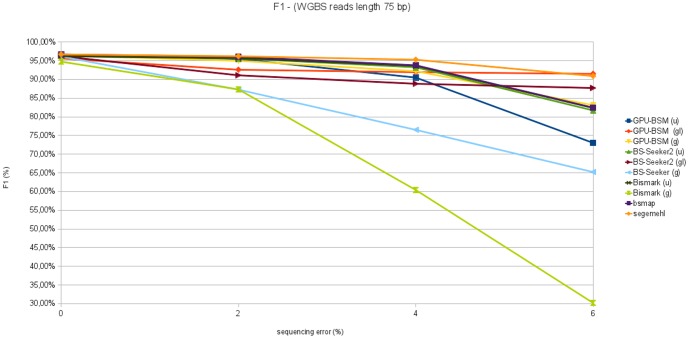
F1 measure analyzing WGBS libraries with reads length of 75 bp. This figure reports F1 measure varying sequencing error from 0% to 6% for 250 thousands of 75 bp reads mapped against the build 37.3 of the human genome.

**Figure 12:**
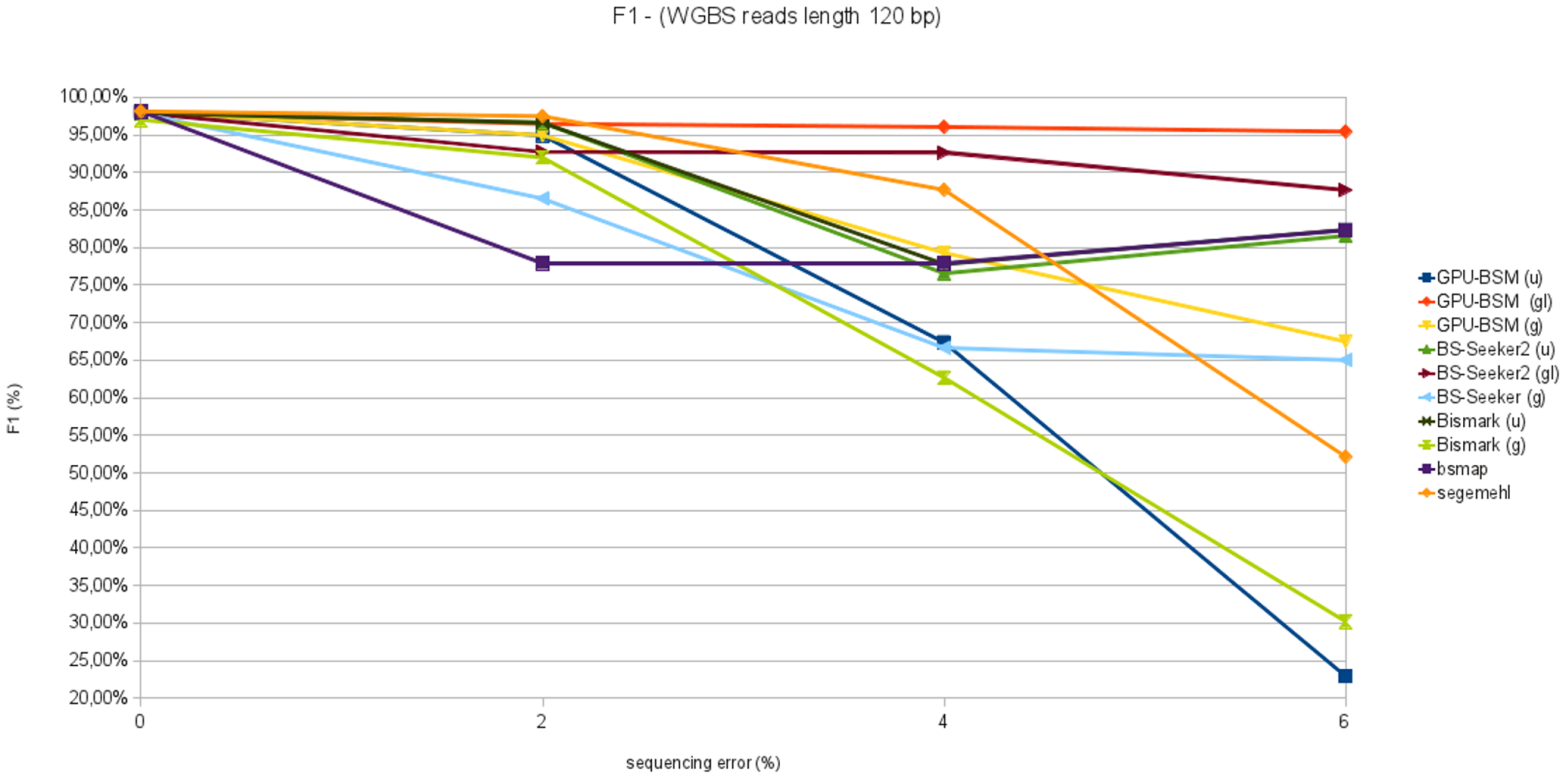
F1 measure analyzing WGBS libraries with reads length of 120 bp. This figure reports F1 measure varying sequencing error from 0% to 6% for 250 thousands of 120 bp reads mapped against the build 37.3 of the human genome.

F1 measures concerning all experiments on WGBS libraries are reported in Figures 11 and 12. These graphs show that GPU-BSM, when run to support local alignments, outperforms BS-Seeker2 for all sequencing errors. As for gapped global alignments, GPU-BSM outperforms Bismark and BS-Seeker2 that exploit the same unbiased strategy, whereas for ungapped alignments its performance is comparable with those of Bismark and BS-Seeker2 only for simulated sequencing error of 0% and 2%.

#### Performance evaluation on RRBS libraries

**Figure 13:**
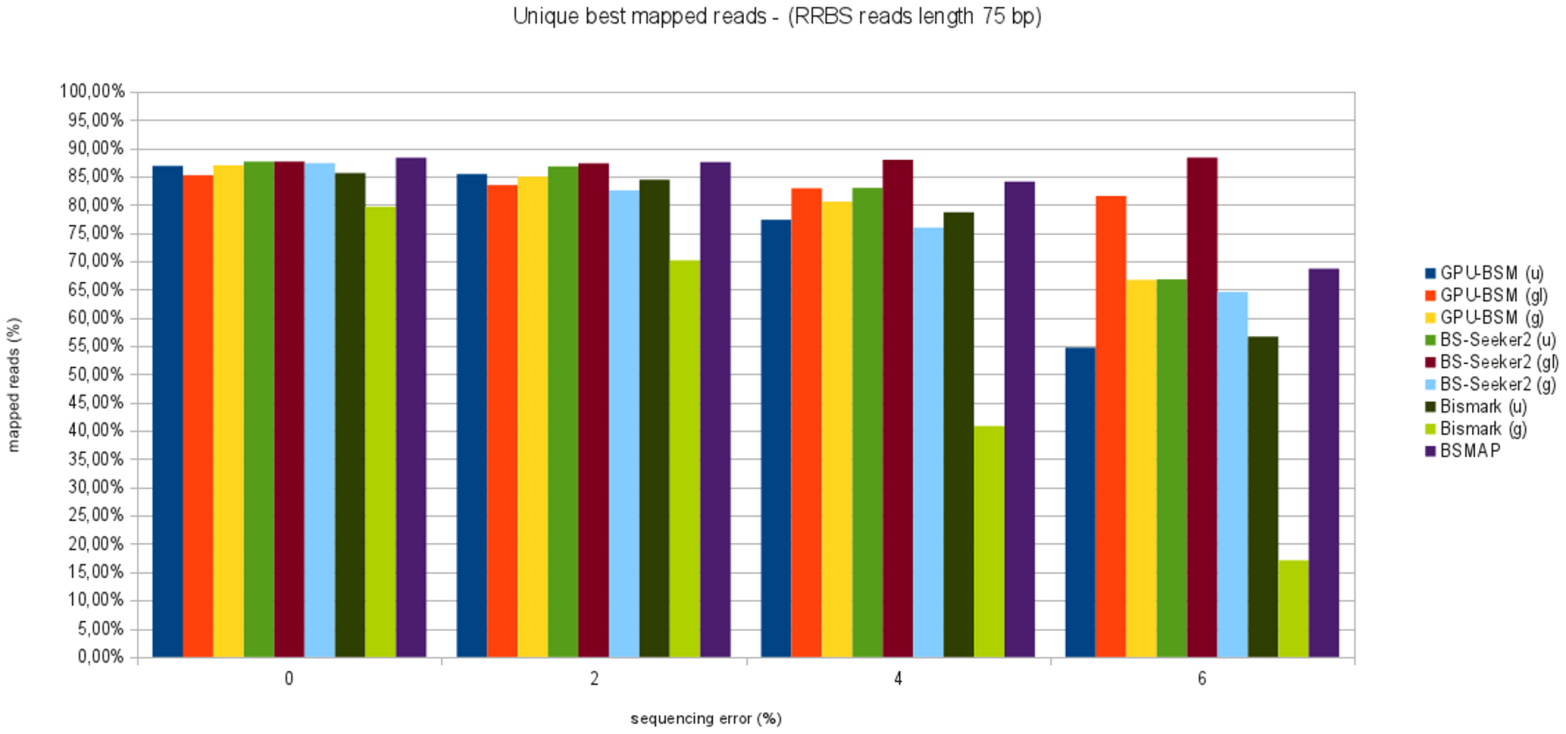
Unique best mapped reads for RRBS libraries with reads length of 75 bp. The graph represents the percentage of unique best mapped reads obtained for each tool as function of the sequencing error for RRBS synthetic libraries with reads length of 75 bp.

**Figure 14:**
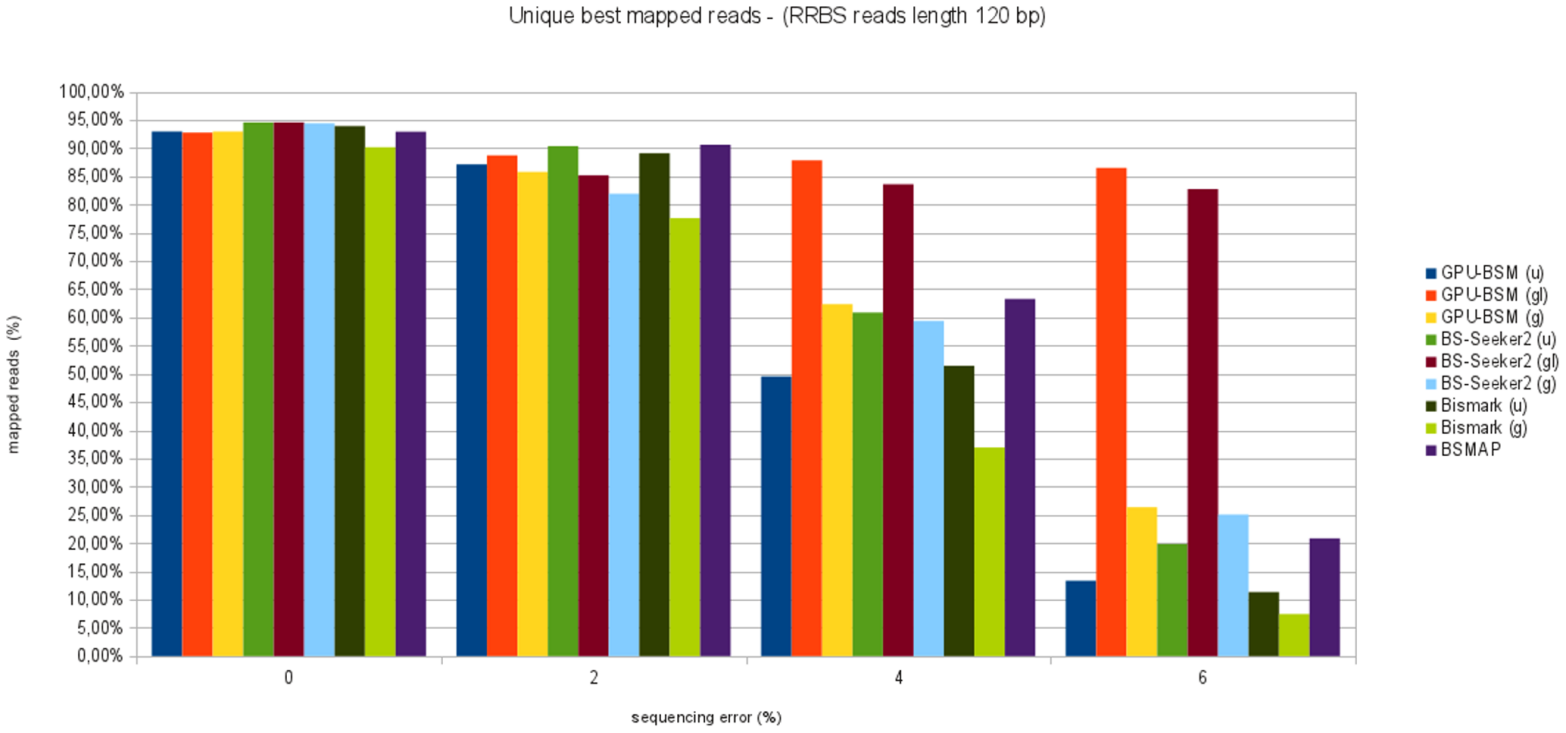
Unique best mapped reads for RRBS libraries with reads length of 120 bp. The graph represents the percentage of unique best mapped reads obtained for each tool as function of the sequencing error for RRBS synthetic libraries with reads length of 120 bp.

Figures 13 and 14 show the percentage of unique best mapped reads as function of sequencing error for RRBS libraries. Also in this case, GPU-BSM and BS-Seeker2, both run to support local alignments, have been able to map more reads than the other tools. As for gapped global alignments, in almost all cases BSMAP has been able to map more reads than the other tools, whereas GPU-BSM and BS-Seeker2 mapped more reads than Bismark. As for ungapped alignments, the performance of GPU-BSM is comparable with those of Bismark and BS-Seeker2 for simulated error sequencing up to 2%. For higher simulated sequencing error BS-Seeker2 mapped more reads than the other tools.

The analysis of precision (see Table 5 and Table 6) shows that GPU-BSM outperforms BS-Seeker2 when run to look for local alignments. As for gapped alignments, BSMAP and Bismark are more accurate than the other tools for reads of length 75 bp and 120 bp respectively. Bismark shows better precision for ungapped alignments.

**Table 5:** Precision for RRBS libraries with reads length of 75 bp.

	simulated sequencing error			
tool	0%	2%	4%	6%
GPU-BSM 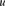	100%	99.26%	98.72%	98.04%
GPU-BSM 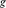	99.98%	99.02%	98.64%	98.64%
GPU-BSM 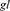	99.95%	98.02%	97.01%	96.27%
Bismark 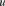	100%	98.96%	98.60%	98.49%
Bismark 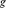	100%	99.43%	98.51%	97.38%
BSMAP	99.92%	99.44%	99.10%	98.48%
BS-Seeker2 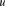	100%	98.72%	97.96%	97.51%
BS-Seeker2 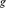	100%	97.41%	96.15%	96.36%
BS-Seeker2 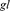	100%	91.42%	87.37%	85.67%

Table reports precision varying the sequencing error from 0% to 6% for 250 thousands of 75 bp reads mapped against the build 37.3 of the human genome.

**Table 6:** Precision for RRBS libraries with reads length of 120 bp.

	simulated sequencing error			
tool	0%	2%	4%	6%
GPU-BSM 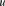	99.27%	98.88%	98.71%	98.32%
GPU-BSM 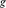	99.26%	99.08%	99.45%	99.45%
GPU-BSM 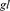	99.24%	98.74%	98.83%	98.68%
Bismark 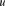	100%	99.72%	99.67%	99.58%
Bismark 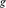	100%	99.79%	99.62%	99.26%
BSMAP	99.91%	99.69%	99.44%	99.06%
BS-Seeker2 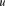	100%	99.66%	99.63%	99.62%
BS-Seeker2 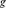	100%	99.04%	99.13%	99.26%
BS-Seeker2 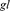	100%	97.14%	96.30%	95.89%

Table reports precision varying the sequencing error from 0% to 6% for 250 thousands of 120 bp reads mapped against the build 37.3 of the human genome.

**Figure 15:**
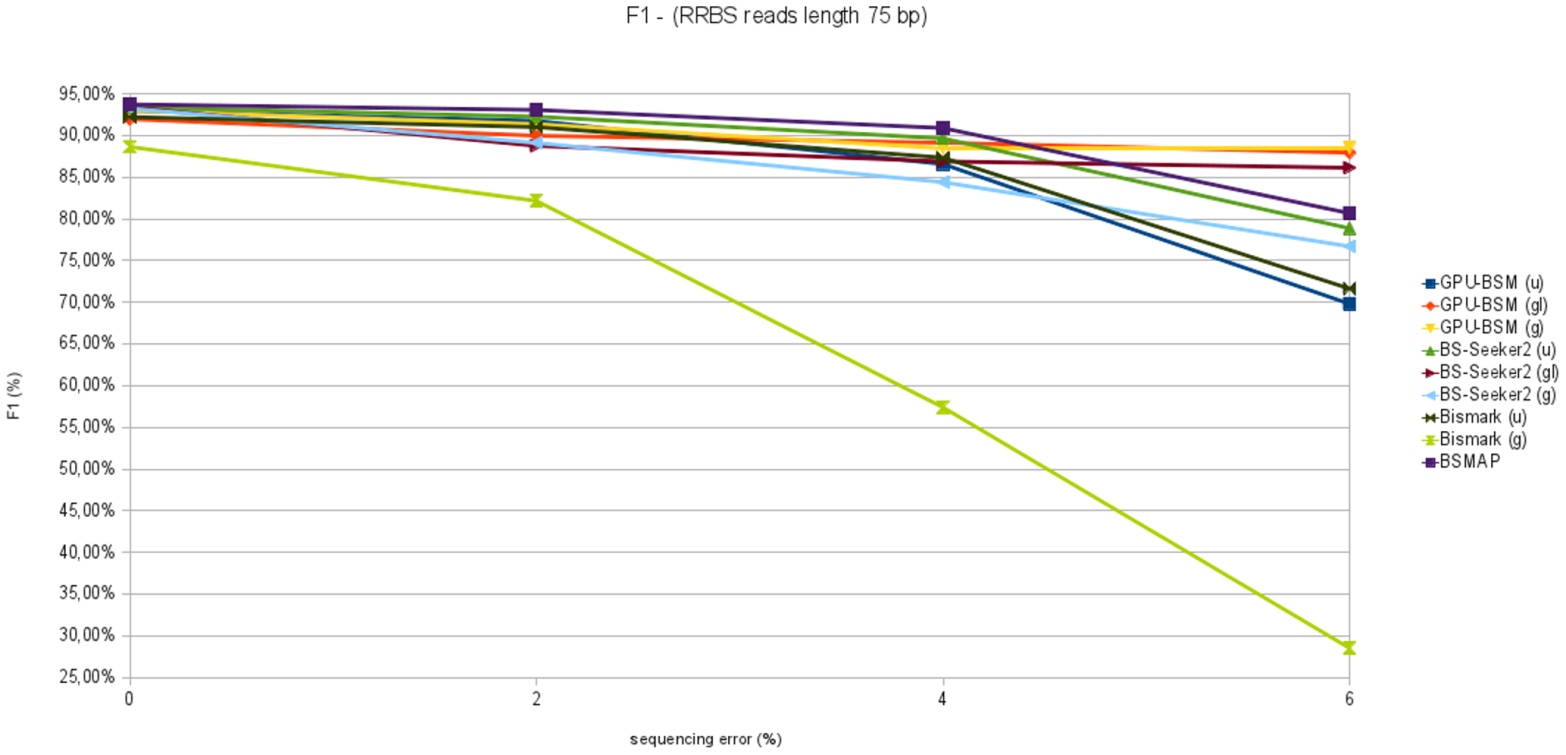
F1 measure analyzing RRBS libraries with reads length of 75 bp. This figure reports F1 measure varying sequencing error from 0% to 6% for 250 thousands of 75 bp reads mapped against the build 37.3 of the human genome.

**Figure 16:**
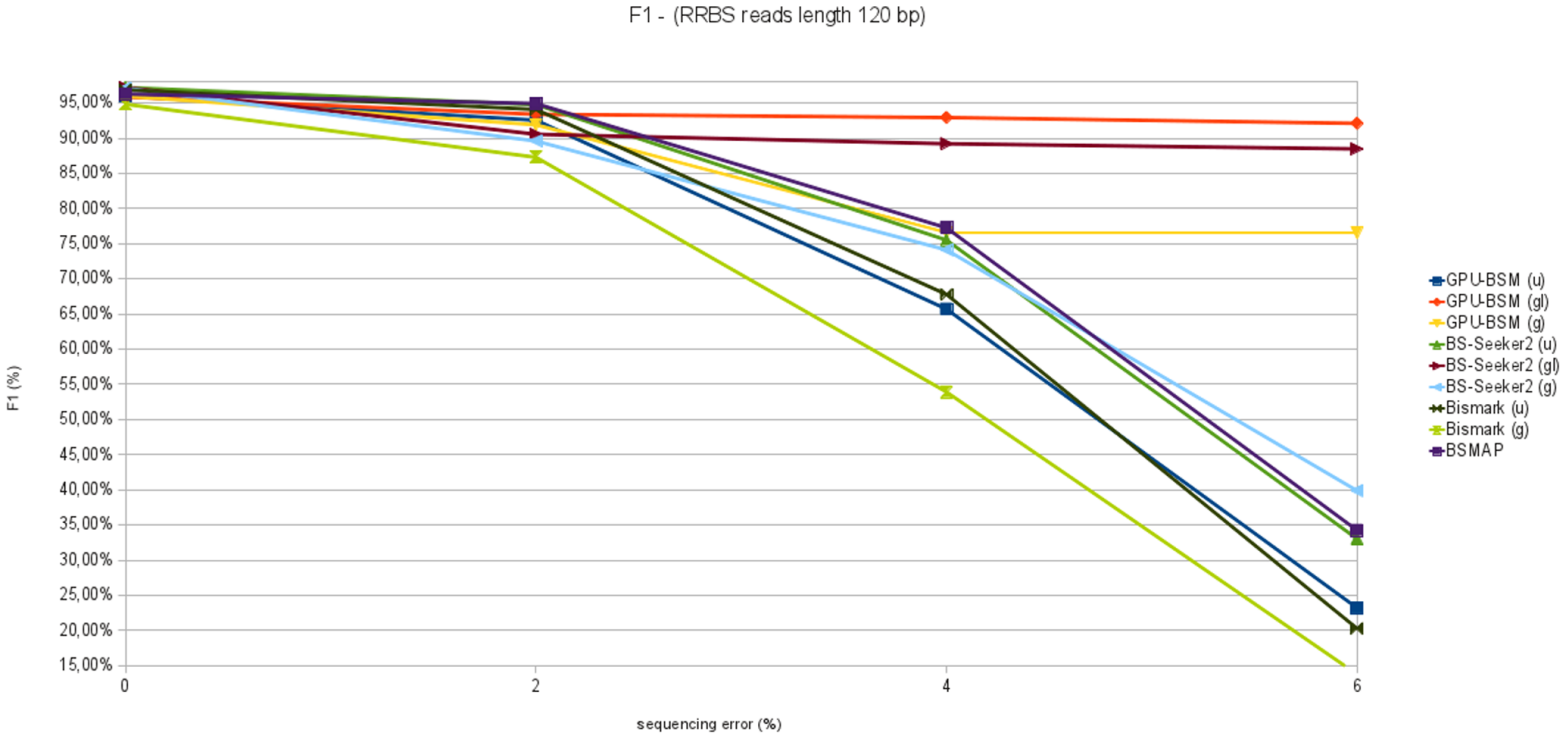
F1 measure analyzing RRBS libraries with reads length of 120 bp. This figure reports F1 measure varying sequencing error from 0% to 6% for 250 thousands of 120 bp reads mapped against the build 37.3 of the human genome.

F1 measures concerning all experiments on RRBS libraries are reported in Figures 15 and 16. These graphs show that GPU-BSM, when run to support local alignments, outperforms BS-Seeker2 for all sequencing errors. As for gapped global alignments BSMAP outperforms the other tools for simulated sequencing error up to 4%. GPU-BSM outperforms the other tools based on the same mapping strategy for all simulated sequencing errors, and BSMAP for simulated errors of 6%. As for ungapped alignments, BS-Seeker2 outperforms all the other tools.

### Performance evaluation on real data

As for WGBS, to assess the performances of GPU-BSM on real data, we used it to map the reads of two directional libraries obtained by sequencing the human H1 cell line on the Human NCBI genome build 37.3/hg19. We mapped the reads of the same real-life libraries analyzed in [14] and [13] to assess the performance of BS-Seeker and segemehl, respectively: library *SRR019597*, consisting of 5.9 millions of 76 bp reads, and library *SRR019048*, consisting of 15.3 millions of 87 bp reads. Results have been compared with those of Bismark, BSMAP, BS-Seeker2, and segemehl.

As for RRBS, we used GPU-BSM to map against the mus musculus genome (mm9) the reads of the library SRR748751 [44]. The library consists of 11.9 millions of 100 bp reads generated with MspI digestion and selecting fragments of 40–220 bp.

Tables 7 and 8 summarize experimental results in terms of fraction of unique best mapped reads and computing time for all tools. In the tables have only been reported the percentage of unique best mapped reads for alignments with up to five differences.

**Table 7:** Performance evaluation on WGBS data.

library	tool	time		percentage of unique best mapped reads					
			differences	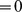	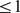	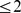	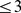	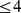	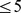
SRR019597	GPU-BSM 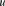	15 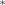 /11 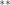 m		40.2%	53.0%	58.8%	62.7%	65.8%	66.6%
	GPU-BSM 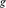	9 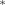 /7 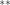 m		40.3%	53.7%	60.1%	64.3%	67.8%	70.9%
	GPU-BSM 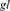	9 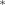 /7 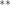 m		61.6%	77.7%	83.5%	86.3%	87.8%	88.7%
	Bismark 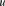	50 m		40.3%	52.8%	58.4%	61.9%	64.1%	65.3%
	Bismark 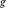	1 h		39.1%	51.5%	56.9%	57.6%	57.8%	57.9%
	BSMAP	13 m		40.0%	52.6%	58.3%	62.0%	64.7%	66.8%
	BS-Seeker2 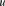	45 m		39.9%	52.3%	58.0%	61.8%	64.7%	67.1%
	BS-Seeker2 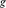	1 h22 m		38.8%	52.5%	56.7%	59.5.0%	61.9%	64.0%
	BS-Seeker2 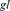	1 h21 m		61.1%	77.8%	83.8%	86.8%	88.6%	89.9%
	Segemehl	32 m		39.9%	53.3%	59.7%	64.2%	67.9%	71.2%
SRR019048	GPU-BSM 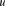	44 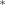 /32 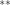 m		24.7%	33.6%	38.0%	41.4%	44.4%	45.1%
	GPU-BSM 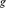	23 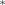 /19 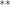 m		24.7%	34.3%	39.1%	42.9%	46.5%	50.0%
	GPU-BSM 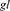	22 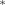 /19 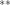 m		55.2%	69.1%	74.0%	76.6%	78.2%	79.3%
	Bismark 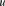	55 m		24.5%	33.3%	37.4%	39.8%	41.2%	42.0%
	Bismark 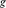	1 h40 m		24.0%	32.7%	36.8%	37.4%	37.7%	37.8%
	BSMAP	1 h30 m		24.6%	33.4%	37.7%	41.0%	43.8%	46.4%
	BS-Seeker2 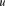	1 h51 m		24.5%	33.2%	37.4%	40.7%	43.6%	46.2%
	BS-Seeker2 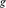	4 h18 m		24.4%	33.2%	37.0%	40.0%	42.9%	45.6%
	BS-Seeker2 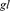	3 h55 m		56.2%	71.5%	77.6%	81.2%	83.7%	85.7%
	Segemehl	57 m		22.8%	33.4%	38.8%	43.2%	47.2%	51.1%

Performances comparison on real-life libraries among GPU-BSM, Bismark, BSMAP, BS-Seeker2, and segemehl. Two directional libraries are analyzed: *SRR019597*, which consists of 5.943.586 reads of length 76 bp, and *SRR019048*, which consists of 15.331.851 reads of length 87 bp. The first and second column of the table report the library and the name of the tools, respectively. The third column reports the time required to analyze the libraries. Columns 4 to 9 report the percentage of uniquely mapped reads according to the number of mapping differences. Differences are mismatches when the tools are used to look for ungapped alignments, whereas they may be mismatches and/or indels when the tools are used to look for gapped alignments. Computing time for GPU-BSM has been reported running it on a single and on two GPUs. As for multi-threading based tools, computing time has been reported for 12 cores. Tools settings: *i*) GPU-BSM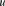 -m 5 –ungapped -l 1, GPU-BSM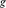 -m 5 –e2e -l 1, GPU-BSM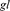 -m 5 -l 1; moreover for all experiments with GPU-BSM the following settings have been used: -L 76 for SRR019597 and -L 87 for SRR019048, -g 0 to run the experiment on a single GPU (-g 0 -g 1 to run the experiment on two GPUs); *ii*) Bismark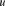 -q ––directional, Bismark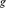 -q –directional –bowtie2 -p 6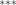; *iii*) BSMAP -v 5 -w 2 -r 0 -p 12; *iv*) BS-Seeker2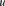 -m 5 –aligner = bowtie -f sam, BS-Seeker2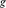 -m 5 –aligner = bowtie2 -f sam –bt2–end-to-end –bt2-p 6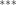, BS-Seeker2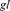 -m 5 –aligner = bowtie2 -f sam –bt2-p 6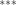 *v*) segemehl -F 1 -H 1 -D 0 -A 70 –threads 12.

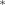
GPU-BSM run on a single GPU.

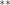
GPU-BSM run on two GPUs.

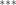
Bismark and BS-Seeker2 run in parallel two instances of Bowtie2. To ensure that both tools use 12 core we used the option -p 6/–bt2-p 6 so that each Bowtie2 instance runs with 6 threads.

**Table 8:** Performance evaluation on RRBS data.

library	tool	time		percentage of unique best mapped reads					
			differences	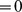	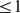	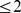	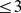	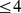	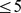
SRR748751	GPU-BSM 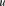	18 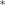 /15 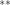 m		24.7%	32.1%	34.6%	35.9%	37.0%	37.1%
	GPU-BSM 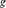	20 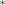 /12 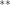 m		24.7%	32.5%	35.1%	35.6%	37.6%	38.3%
	GPU-BSM 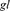	27 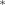 /18 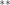 m		41.8%	50.1%	52.4%	53.4%	54.0%	54.3%
	Bismark 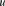	34 m		24.2%	31.1%	33.2%	33.9%	34.3%	34.6%
	Bismark 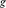	56 m		22.3%	28.9%	31.0%	32.2%	32.5%	32.6%
	BSMAP	6 m		24.6%	31.6%	33.7%	34.9%	35.7%	36.3%
	BS-Seeker2 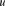	52 m		24.6%	31.7%	33.7%	34.8%	35.5%	35.9%
	BS-Seeker2 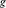	4 h6 m		24.5%	31.8%	34.2%	35.6%	36.7%	37.7%
	BS-Seeker2 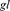	3 h29 m		48.9%	59.5%	63.4%	65.7%	67.0%	67.9%

Performances comparison on real-life libraries among GPU-BSM, Bismark, BSMAP, and BS-Seeker2. A directional library *SRR748751*, which consists of 11.961.710 reads of length 100 bp has been analyzed. The first and second column of the table report the library and the name of the tools, respectively. The third column reports the time required to analyze the library. Columns 4 to 9 report the percentage of uniquely mapped reads according to the number of mapping differences. Differences are mismatches when the tools are used to look for ungapped alignments, whereas they may be mismatches and/or indels when the tools are used to look for gapped alignments. Computing time for GPU-BSM has been reported running it on a single and on two GPUs. As for multi-threading based tools, computing time has been reported for 12 cores. Tools settings: *i*) GPU-BSM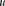 -m 5 –ungapped -l 1 -R -d C-CGG, GPU-BSM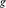 -m 5 –e2e -l 1 -R -d C-CGG, GPU-BSM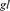 -m 5 -l 1 -R -d C-CGG; moreover for all experiments with GPU-BSM the following setting has been used: -L 100 -g 0 to run the experiment on a single GPU (-L 100 -g 0 -g 1 to run the experiment on two GPUs); *ii*) Bismark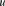 -q –directional, Bismark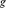 -q –directional –bowtie2 -p 6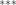; *iii*) BSMAP -v 5 -w 2 -r 0 -D -C-CGG -p 12; *iv*) BS-Seeker2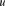 -m 5 –aligner = bowtie -f sam -r -c C-CGG -L 40 -U 220, BS-Seeker2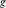 -m 5 –aligner = bowtie2 -f sam -r -c C-CGG -L 40 -U 220 –bt2–end-to-end –bt2-p 6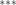, BS-Seeker2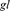 -m 5 –aligner = bowtie2 -f sam -r -c C-CGG -L 40 -U 220 –bt2-p 6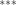.

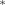
GPU-BSM run on a single GPU.

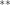
GPU-BSM run on two GPUs.

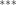
Bismark and BS-Seeker2 run in parallel two instances of Bowtie2. To ensure that both tools use 12 core we used the option -p 6/–bt2-p 6 so that each Bowtie2 instance runs with 6 threads.

Experimental results show that GPU-BSM is a very effective tool for mapping bisulfite-treated reads, as it outperforms almost all analyzed tools. When run to look for ungapped and gapped global alignments, it has been able to map more reads than the other tools in almost all cases. As for unique best mapped reads, its performances are only comparable with those of segemehl for WGBS libraries. GPU-BSM appears to be slightly more effective than segemehl to map reads with few differences. On the other hand segemehl appears to be slightly more effective to map reads with more differences. When run to look for local alignment BS-Seeker2 mapped more reads than GPU-BSM.

As for the computing time, GPU-BSM is definitely the faster tool to map WGBS libraries, and the second to map RRBS libraries. In particular, as for SRR0195957/SRR019048 WGBS libraries, GPU-BSM ran on a single GPU resulted: *i*) 3.3x/1.25x faster than Bismark and 3x/2.5x faster than BS-Seeker2 when run to look for ungapped alignments; *ii*) 6.6x/4.3x faster than Bismark, 9x/11.2x faster than BS-Seeker2, 3.5x/2.4x faster than segemehl, and 1.4x/3.9x faster than BSMAP when run to look for gapped global alignments; *iii*) 9x/10.6x faster than BS-Seeker2 to map reads with gapped local alignments. As for the SRR748751 RRBS library, GPU-BSM ran on a single GPU resulted: *i*) 1.9x faster than Bismark and 2.8x faster than BS-Seeker2 when run to look for ungapped alignments; *ii*) 2.8x faster than Bismark and 12.3x faster than BS-Seeker2 when run to look for gapped global alignments; *iii*) 7.7x faster than BS-Seeker2 to map reads with gapped local alignments. As for RRBS and gapped global alignments, BSMAP resulted 3.3x faster than GPU-BSM.

### Hardware and Software Configuration

Experiments described hereinafter have been carried out on a 12 cores Intel Xeon CPU E5-2667 2.90 GHz with 128 GB of RAM. Two NVIDIA Kepler architecture based Tesla k20c cards with 0.71 GHz clock rate and equipped with 4.8 GB of global memory have been exploited to execute SOAP3-dp rel. 2.3.177.

## Discussion

GPU-BSM is a mapping tool able to align single-end and paired-end reads generated from WGBS and RRBS. GPU-BSM supports both gapped and ungapped alignments. Massive parallelization on GPUs enables GPU-BSM to map reads without stringent limitations on the alignment process. Experimental results shown that GPU-BSM is very accurate and outperforms most of the cutting-edge solutions in terms of unique best mapped reads, while keeping computational time reasonably low.

We deem there are further margins of improvement of the overall computing time. The mapping process implemented in GPU-BSM can be represented by a three-stage pipeline. In the first stage, GPU-BSM performs a 3-letter nucleotide alphabet reduction. Successively, the bisulfite-treated reads are mapped against the reference genome. Finally, GPU-BSM analyzes the mapped reads to detect and remove those ambiguous and false positives. Currently, only the second stage of the pipeline has been parallelized on GPU cards. In particular, the mapping process can be run on up to four GPU cards. At the second stage, the gain in terms of computing time resulted nearly linear with increasing the number of GPU cards. Nevertheless, the overall gain is not linear due to the fact that the first and third stages of the pipeline have not yet been parallelized. We are working to improve GPU-BSM *i*) porting to GPU the third stage of the pipeline and *ii*) extending the parallelization of the second stage to a cluster of GPUs. Porting to GPUs the analysis performed at the third stage is essential to obtain a linear gain of the computing time with increasing the used GPUs. Without this improvement, there will be no benefit from the parallelization of the second stage on a cluster of GPUs. We estimated that the planned updates of GPU-BSM can notably improve the computing time. We implemented this part of the algorithm with the aim to easily migrate it on GPU. In doing this, we defined data structures mainly devised for massive parallelization on GPU that are not optimized for CPU. This implied a huge amount of memory required to run it. We reported the peaks of memory required from the different tools in Table 9 which shows that only segemehl requires more memory than GPU-BSM.

**Table 9:** Memory consumption.

library	tool	memory
SRR019597	GPU-BSM 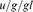	20.3/20.3/20.3 GB
	Bismark 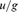	7.7/10.1 GB
	BSMAP	8.3 GB
	BS-Seeker2 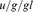	4.6/7.3/7.3 GB
	segemehl	53 GB
SRR019048	GPU-BSM 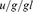	22.4/40.6/41.6 GB
	Bismark 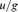	7.7/10.1 GB
	BSMAP	8.3 GB
	BS-Seeker2 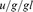	4.6/7.3/7.3 GB
	segemehl	53 GB
SRR748751	GPU-BSM 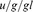	17.3/27.7/29.5 GB
	Bismark 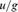	7.7/10.1 GB
	BSMAP	2.1 GB
	BS-Seeker2 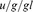	3.0/3.0/3.0 GB

Peaks of memory required to run experiments on real-life libraries. Data reported in the table shows that GPU-BSM is not very efficient in terms of memory consumption.

GPU-BSM is freely available for non-commercial use under the terms of the Affero GNU General Public License. The current release can be downloaded at the following addresses http://pypi.python.org/pypi/GPU-BSM/ and http://www.itb.cnr.it/web/bioinformatics/gpu-bsm.
